# Detection of genome-edited cells by oligoribonucleotide interference-PCR

**DOI:** 10.1093/dnares/dsy012

**Published:** 2018-04-27

**Authors:** Toshitsugu Fujita, Miyuki Yuno, Fusako Kitaura, Hodaka Fujii

**Affiliations:** 1 Department of Biochemistry and Genome Biology, Hirosaki University Graduate School of Medicine, Hirosaki, Aomori, Japan; 2 Chromatin Biochemistry Research Group, Combined Program on Microbiology and Immunology, Research Institute for Microbial Diseases, Osaka University, Suita, Osaka, Japan

**Keywords:** PCR, ORNi-PCR, genome editing

## Abstract

Genome editing by engineered sequence-specific nucleases, such as the clustered regularly interspaced short palindromic repeats (CRISPR) system is widely used for analysis of gene functions. Several techniques have been developed for detection of genome-edited cells, but simple, cost-effective, and positive detection methods remain limited. Recently, we developed oligoribonucleotide (ORN) interference-PCR (ORNi-PCR), in which hybridization of an ORN with a complementary DNA sequence inhibits amplification across the sequence. Here, we investigated whether ORNi-PCR can be used to detect genome-edited cells. First, we showed that ORNs that hybridize to a CRISPR target site in the *THYN1* locus inhibited amplification across the target site, but no longer inhibited amplification after the target site was edited, resulting in mismatches. Importantly, ORNi-PCR could distinguish even single-nucleotide differences. These features of ORNi-PCR enabled detection of genome-edited cells by positive PCR amplification. In addition, ORNi-PCR was successful in discriminating genome-edited cells from wild-type cells, and multiplex ORNi-PCR simultaneously detected indel mutations at multiple loci. However, endpoint ORNi-PCR may not be able to distinguish between mono- and bi-allelic mutations, which may limit its utility. Taken together, these results demonstrate the potential utility of ORNi-PCR for the screening of genome-edited cells.

## 1. Introduction

Genome editing is an essential biotechnology for medical and biological research. Zinc finger nucleases (ZFNs), transcription activator-like effector nucleases (TALENs), and the clustered regularly interspaced short palindromic repeats (CRISPRs) system have been widely used as genome-editing tools.^1–3^ Because CRISPR is the most convenient tool, it is rapidly becoming the predominant technique for genome editing.^1–3^ CRISPR also makes it much easier to perform genome editing in various cell types.^3^

For such approaches to be successful, screening methods capable of positively identifying genome-edited cells are indispensable. Although DNA sequencing can unambiguously detect genome-edited cells, it is time-consuming, costly, and unsuitable for initial screening of a large number of clones. Therefore, various methods have been developed for rapid and inexpensive detection of the desired cells.^4^ For example, mismatch cleavage assays using T7 endonuclease 1 (T7E1) or Surveyor nuclease have been used to evaluate mutation frequency. In these techniques, following PCR amplification of a DNA sequence spanning a CRISPR target site, the amplicons are denatured, and re-hybridized to form heteroduplexes of wild-type (WT) and mutated strands. Mismatches between edited and unedited sites in the heteroduplexes are cleaved by T7E1 or Surveyor nuclease, and the resultant DNA fragments are electrophoresed to assess mutation frequency. The re-hybridized DNA strands can also be directly analysed by native polyacrylamide gel electrophoresis.^5^ It is also possible to analyse mobility of the amplicons of WT and mutated strands by capillary electrophoresis.^6^^,^^7^ Real-time PCR has also been used to evaluate the success of genome editing. For example, in high-resolution melting analysis, melting curves of PCR amplicons are analysed to distinguish nucleotide-level differences.^8^ Alternatively, by monitoring fluorescence emission of SYBR Green, real-time PCR with a primer designed against the target site can be used to assess the presence of indel mutations.^9^ In this approach, inhibition of PCR amplification indicates introduction of indel mutations into the primer-binding site (i.e. the target site). Real-time PCR with fluorescent probes complementary to the target site (e.g. TaqMan probes) can also be used to assess the presence of indel mutations.^10^ Digital PCR with complementary fluorescent probes can be used for more accurate analysis.^10–12^ Although those methods are useful for detecting genome-edited cells, some require expensive equipment, such as real-time PCR machines or capillary gel electrophoresis systems, and they are also time-consuming. To address this issue, simpler methods using endpoint PCR were developed,^13^^,^^14^ in which a primer designed against the target site is used in nearly the same way as in the real-time PCR-based method.^9^ Endpoint PCR-based approaches can also be more cost-effective for the evaluation of indel mutations.

**Figure 1.**
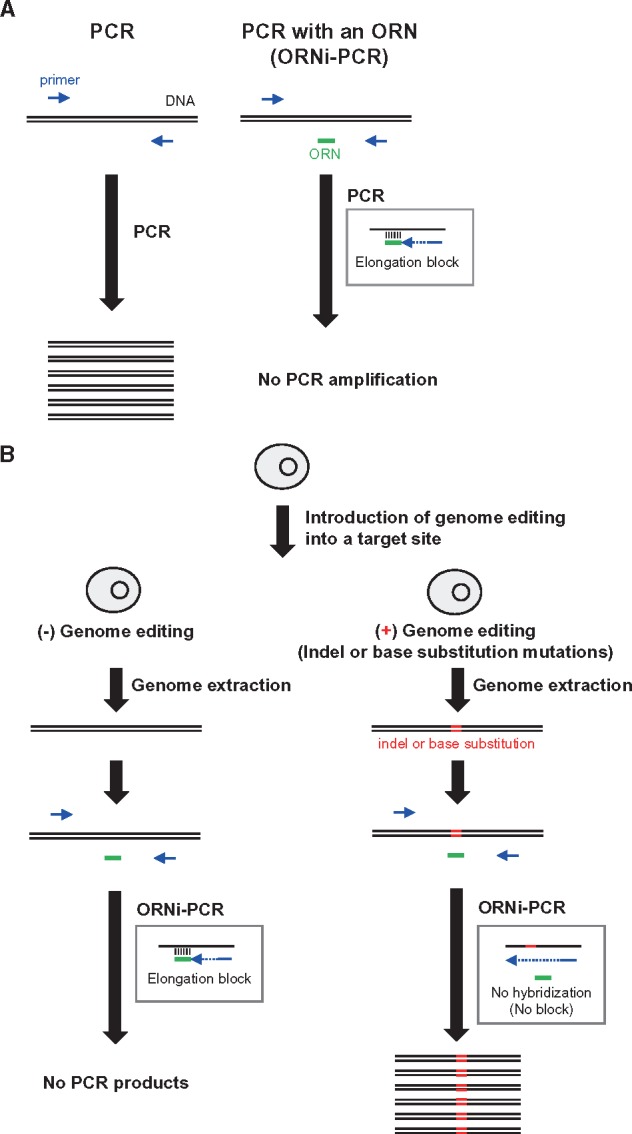
Detection of genome-edited cells by ORNi-PCR. (A) Schematic of ORNi-PCR. An ORN [not ODN] inhibits PCR amplification of a target DNA sequence that contains the DNA sequence complementary to the ORN. (B) Application of ORNi-PCR to detection of genome-edited cells.

We recently developed oligoribonucleotide (ORN) interference-PCR (ORNi-PCR) to inhibit PCR amplification in a sequence-specific manner (Fig. 1A).^15^ In ORNi-PCR, an ORN [not an oligodeoxyribonucleotide (ODN)] with a length of 17–29 bases inhibits PCR amplification of a target DNA sequence that contains the DNA sequence complementary to the ORN (Fig. 1A). We hypothesized that ORNi-PCR could be applied to detection of genome-edited cells (Fig. 1B). In this approach, an ORN is designed to hybridize with the target site for genome editing. Next, PCR is performed in the presence of the ORN on genomic DNAs (gDNAs) extracted from the genome-edited cells. If a mutation has been successfully introduced into the target site, the ORN fails to hybridize, resulting in amplification of the target DNA. In other words, positive PCR amplification indicates that the target site is mutated, whereas no PCR amplification indicates that the target site is intact (Fig. 1B). Because chemical synthesis of ORNs is inexpensive, it is possible to screen economically a large number of potential genome-edited cells by ORNi-PCR. In addition, endpoint ORNi-PCR does not require expensive equipment such as real-time PCR machines.

In this study, we investigated whether ORNi-PCR can be used for detection of genome-edited cells. We demonstrated that ORNi-PCR can distinguish indel mutations with even a single-nucleotide difference relative to the intact DNA sequences of target sites. However, we also found that it may be difficult to distinguish between mono- and bi-allelic mutations by endpoint ORNi-PCR. Thus, the primary use of ORNi-PCR could be the detection of genome-edited cells possessing at least mono-allelic mutations at a target site before subsequent determination of mono- or bi-allelic mutations by other methods.

## 2. Materials and methods

### 2.1 Oligonucleotides

The primers used in this study are listed in Supplementary Table S1. ORNs were chemically synthesized (Greiner) and are listed in Supplementary Table S2.

### 2.2 Cell culture and extraction of gDNAs

Raji cells were cultured in RPMI-1640 (Wako) supplemented with 10% foetal bovine serum (FBS). 293T cells were cultured in Dulbecco's modified Eagle medium (DMEM) (Wako) supplemented with 10% FBS. HCT116 cells were cultured in McCoy’s 5A (Thermo Fisher Scientific) supplemented with 10% FBS. gDNAs were extracted from cells by standard phenol/chloroform extraction.

### 2.3 ORNi-PCR

ORNi-PCR reactions were performed using KOD-Plus-Ver.2 (Toyobo). For ORNi-PCR targeting the human *THYN1* locus, ORNi-PCR reaction mixtures containing 20 ng of Raji gDNA, 0.3 µM of each primer, and 0.1–2 µM of an ORN were prepared in a 10 μl volume according to the manufacturer’s protocol. The reactions were carried out with an initial denaturation at 94°C for 2 min, followed by 35 cycles of 98°C for 10 s, 62°C for 30 s, and 68 °C for 1 min. For ORNi-PCR targeting the human *CDKN2A(p16)* locus, ORNi-PCR reaction mixtures containing 20 ng of 293T or HCT116 gDNA, 0.3 µM of each primer, and 1 µM of an ORN were prepared in a 10 μl volume. The reactions were carried out with an initial denaturation at 94°C for 2 min, followed by 30 cycles of 98°C for 10 s, 62°C for 30 s, and 68°C for 1 min (for Fig. 6), or an initial denaturation at 94°C for 2 min, followed by 30 cycles of 98°C for 10 s, and 62–72°C for 20 s (for Figs 8 and 9). For ORNi-PCR targeting both the human *THYN1* and *CDKN2A(p16)* loci, ORNi-PCR reaction mixtures containing 20 ng of 293T gDNA, 0.3 µM of each primer, and 1 µM of each ORN were prepared in a 10 μl volume. The reactions were carried out with an initial denaturation at 94°C for 2 min, followed by 30 cycles of 98°C for 10 s, 62°C for 30 s, and 68°C for 1 min. ORNi-PCR products were electrophoresed on 1% or 2% agarose gels and, if necessary, subjected to DNA sequencing. DNA-sequencing data were analysed using the Applied Biosystems Sequence Scanner Software v2.0 (Thermo Fisher Scientific).

### 2.4 Plasmids

The Cas9 expression plasmid (Addgene no. 41815)^16^ and chimeric single guide RNA (sgRNA) expression plasmid (Addgene no. 41824)^16^ were provided by Dr George Church through Addgene. To construct a sgRNA expression plasmid targeting the human *THYN1* locus, a CRISPR target sequence was cloned downstream of the U6 promoter in the sgRNA expression plasmid according to the hCRISPR gRNA synthesis protocol (https://media.addgene.org/data/93/40/adf4a4fe-5e77-11e2-9c30-003048dd6500.pdf (19 April 2018, date last accessed)). To construct a Cas9 plus sgRNA expression plasmid targeting the human *CDKN2A(p16)* locus, the sgRNA expression cassette for *CDKN2A(p16)* (Gx4 no. 2)^17^ was cloned upstream of the Cas9 expression cassette in the Cas9 expression plasmid.

### 2.5 CRISPR-mediated genome editing

For genome editing of the human *THYN1* locus, Raji cells (1 × 10^7^) were transfected with Cas9 expression plasmid (120 µg), sgRNA expression plasmid targeting the human *THYN1* locus (120 µg), and pEGFP-N3 (0.3 µg, Clontech) by electroporation on a Gene Pulser II (Bio-Rad) at 250 V and 950 µF. One day later, GFP-positive cells were individually sorted and expanded. For genome editing of the human *CDKN2A(p16)* locus, 293 T cells (4 × 10^5^) were transfected with the Cas9 plus sgRNA expression plasmid targeting the human *CDKN2A(p16)* locus (4 µg) and pcDNA3.1/Hygro (–) (0.4 µg, Thermo Fisher Scientific) using Lipofectamine 3000 (Thermo Fisher Scientific). Two days later, hygromycin was added (0.4 mg/ml), and hygromycin-resistant colonies were picked and cultured. For genome editing of the human *THYN1* and *CDKN2A(p16)* loci, 293 T cells (4 × 10^5^) were transfected with the Cas9 plus sgRNA expression plasmid targeting the human *CDKN2A(p16)* locus (4 µg), sgRNA expression plasmid targeting the human *THYN1* locus (4 µg), and pcDNA3.1/Hygro (−) (0.4 µg) using Lipofectamine 3000. Two days later, hygromycin was added (0.4 mg/ml), and hygromycin-resistant colonies were picked and cultured.

## 3. Results

### 3.1 Determination of effective concentrations of ORNs for ORNi-PCR

**Figure 2.**
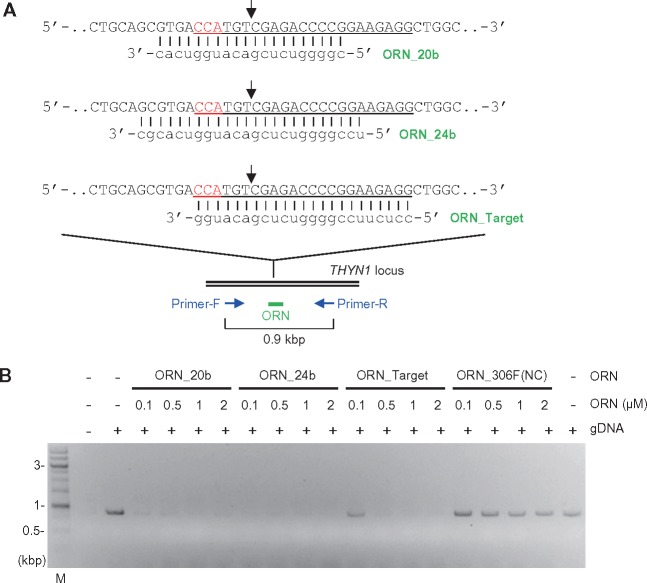
Suppression of PCR amplification of a target DNA sequence by ORNs. (A) Target positions and sequences of ORNs. The forward DNA sequence of the allele is shown. CRISPR target sites are underlined, and PAM positions are shown in red. Black arrows indicate the CRISPR cleavage sites. (B) Dose responses of ORNs. PCR amplification was performed in the presence or absence of various concentrations of ORNs. M, molecular weight marker.

First, we asked whether ORNi-PCR could suppress amplification across a CRISPR target site in the human *THYN1* locus. We designed three ORNs: ORN_20b, ORN_24b, and ORN_Target. ORN_20b (20 bases) and ORN_24b (24 bases) hybridize with the target site such that the CRISPR cleavage position, which is 3 bp upstream of the protospacer adjacent motif (PAM),^18^ is in the centre of their sequences. ORN_Target (23 bases) matches the 20 bp sequence of the sgRNA as well as the PAM sequence used for genome editing (Fig. 2A). Because DNA polymerases that retain 3′–5′ exonuclease activity, but not those that retain 5′–3′ exonuclease activity, can be utilized for ORNi-PCR,^15^ we used the KOD DNA polymerase^19^ to amplify a 0.9 kb region surrounding the target site (Fig. 2A). As shown in Fig. 2B, the 0.9 kb region was specifically amplified when gDNA extracted from human Raji cells was used for PCR in the absence of ORNs. Addition of ORN_20b (1 or 2 μM), ORN_24b (0.1–2 μM), or ORN_Target (0.5–2 μM) to the reactions strongly suppressed PCR amplification. Lower concentrations of ORN_20b (0.1 or 0.5 μM) and ORN_Target (0.5 µM) were less effective in this respect. In contrast, ORN_306F(NC), a 25-base ORN hybridizing with an irrelevant locus (human *IRF-1* locus),^15^ did not affect amplification (Fig. 2B). Thus, these *THYN1*-specific ORNs can be used to specifically suppress PCR amplification of the human *THYN1* locus.

### 3.2 Detection of genome-edited cells using ORNi-PCR

**Figure 3.**
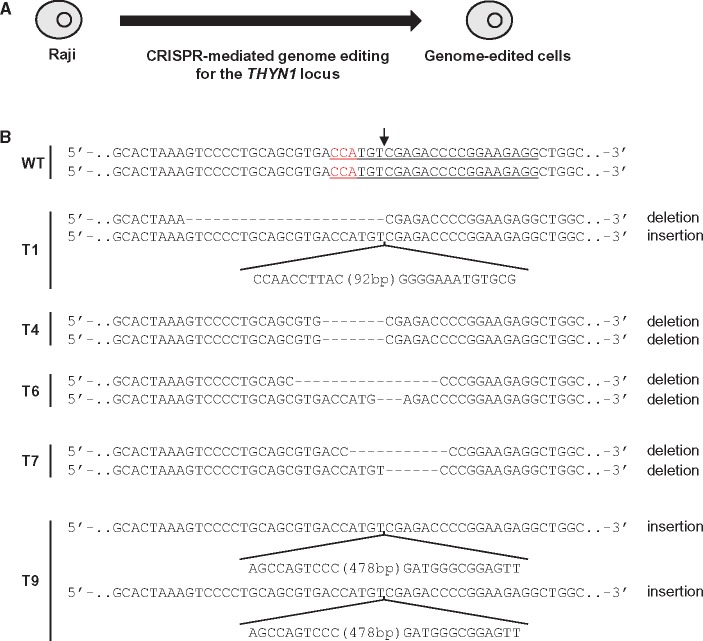
Indel mutations introduced by CRISPR-mediated genome editing. (A) Schematic of CRISPR-mediated genome editing in Raji cells. (B) DNA sequences around the CRISPR target site in the *THYN1* locus in WT and genome-edited cells. The forward DNA sequences of both alleles are shown. The CRISPR target sites in WT are underlined, and PAM positions are shown in red. An arrow indicates the CRISPR cleavage sites.

**Figure 4.**
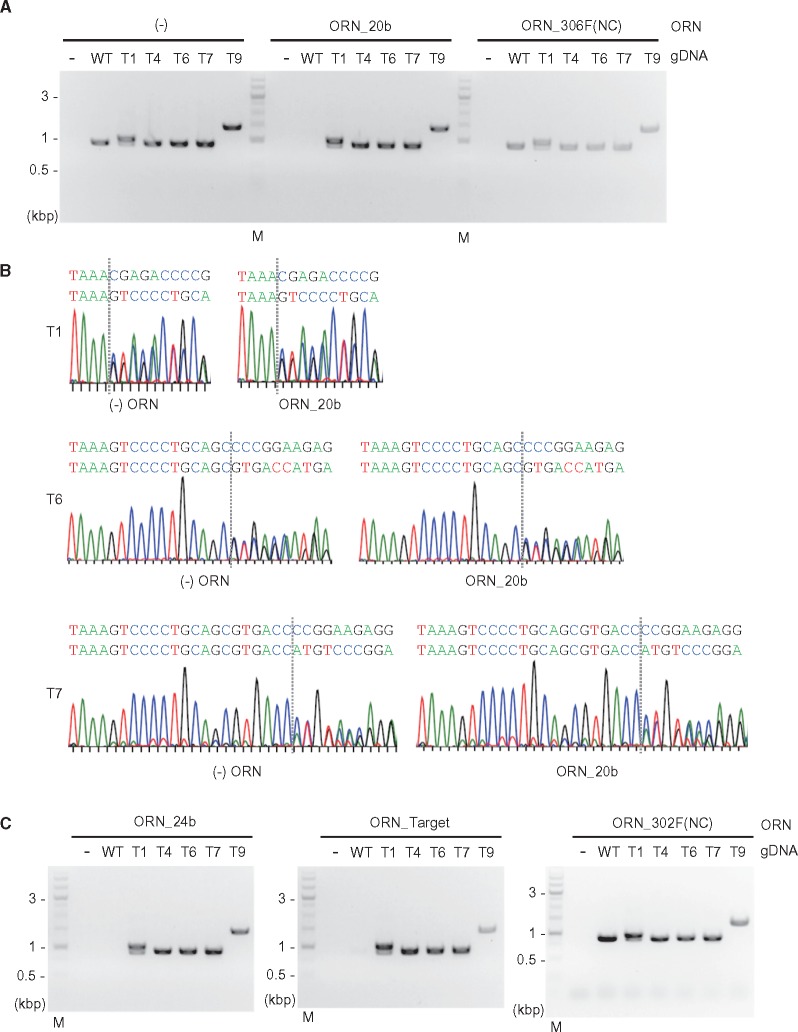
Detection of bi-allelic genome editing by ORNi-PCR. (A and C) ORNi-PCR with gDNAs extracted from WT and genome-edited Raji cells. PCR was performed in the presence (1 µM) or absence of ORNs. M: molecular weight marker. (B) DNA-sequencing signals of PCR products. PCR products of T1, T6, and T7 in (A) [(−) ORN and ORN_20b] were purified from agarose gels and subjected to DNA sequencing.

To demonstrate that this approach can detect genome-edited cells (Fig. 1B), we next investigated whether ORNs could suppress PCR amplification across an intact CRISPR target site, but not an edited variant of the same site. To this end, we performed CRISPR-mediated genome editing of the *THYN1* locus in the Raji line and established genome-edited clones in which the target site was mutated in both alleles (Fig. 3A). The genome-edited cells contained indel mutations in the range of 3–501 bp (Fig. 3B and Supplementary Figs S1 and S2). Clone T6 possesses the shortest indel mutation (3 bp deletion) in one allele, whereas T9 has the longest indel (501 bp insertion) in both alleles. T4 and T9 harboured homozygous indel mutations. PCR with the *THYN1*-specific primer set (Fig. 2A) amplified the target region from gDNAs of all cells in the absence of ORNs (Fig. 4A). In contrast, ORN_20b completely suppressed the PCR amplification from gDNA of WT Raji but not the genome-edited cells (Fig. 4A). ORN_306F(NC) had no effect on amplification.

Because ORN_20b might allele-specifically suppress PCR amplification from gDNAs of T1, T6, and T7 cells, in which the CRISPR target site is differentially mutated in each allele (Fig. 3B), we sequenced the ORNi-PCR product. As shown in Fig. 4B, the sequencing signals of the ORNi-PCR products were comparable to those of PCR products generated in the absence of an ORN [(−) ORN], demonstrating that ORN_20b did not affect amplification from those gDNAs. Next, we tested other ORNs. ORNi-PCR with ORN_24b or ORN_Target, but not ORN_302F(NC), another irrelevant 21-base ORN,^15^ yielded PCR patterns identical to those of ORNi-PCR with ORN_20b (Fig. 4C and Supplementary Fig. S3). Thus, these results showed that ORNi-PCR can be used to distinguish bi-allelic indel mutations from an intact target sequence. Amplification of T6 gDNA was not affected by the *THYN1*-specific ORNs, indicating that ORNi-PCR can distinguish indel mutations of more than 3 bp in size.

**Figure 5.**
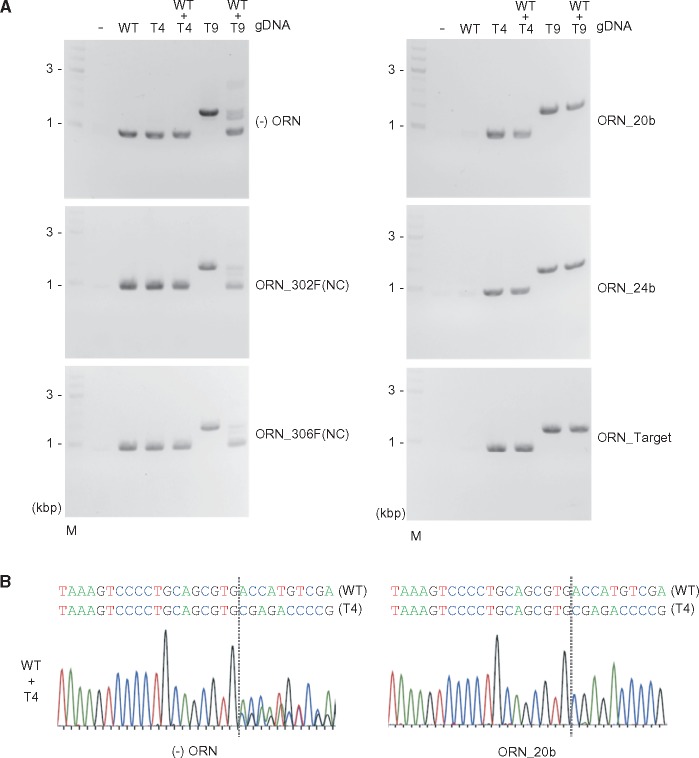
Detection of mono-allelic genome editing by ORNi-PCR. (A) ORNi-PCR with gDNAs extracted from WT and genome-edited Raji cells. To mimic mono-allelic mutations, gDNAs of WT and T4 or T9 were mixed at 1:1 ratio. PCR was performed in the presence (1 µM) or absence of ORNs. M: molecular weight marker. (B) DNA-sequencing signals of PCR products. PCR products of WT + T4 in (A) [(−) ORN and ORN_20b] were purified from agarose gels and subjected to DNA sequencing.

Next, we sought to determine whether ORNi-PCR can also distinguish mono-allelic mutations from an intact target sequence. To this end, we mixed gDNAs extracted from WT and T4 or T9 cells at 1:1 ratio to mimic mono-allelic mutations (Fig. 5). When the mixture of WT and T9 gDNAs was used in ORNi-PCR with ORN_20b, ORN_24b, or ORN_Target, PCR amplification was suppressed only when the samples contained WT gDNA, but not mutated gDNA (Fig. 5A). When the same experiment was performed with the mixture of WT and T4 gDNAs, a single band (0.9 kb) was detected (Fig. 5A); DNA sequencing of the ORNi-PCR product using ORN_20b detected the signal derived from T4 but not WT (Fig. 5B), clearly showing that ORNi-PCR amplified the edited genomic region but not the intact site. We also obtained the same results using ORNi-PCR with other *THYN1*-specific ORNs (Supplementary Fig. S4). Thus, these results demonstrated that ORNi-PCR can be used to distinguish mono-allelic indel mutations from an intact target sequence.

Taken together, these findings show that ORNi-PCR is a useful method for distinguishing mono- and bi-allelic indel mutations from an intact target sequence. However, endpoint ORNi-PCR may not be suitable to discriminate mono- from bi-allelic indel mutations, largely because it may be difficult to quantitatively distinguish PCR products amplified from one- and two-copy templates.

### 3.3 ORNi-PCR using the *Pfu* DNA polymerase, a real-time PCR machine, and crRNAs

The *Pfu* DNA polymerase retains 3′–5′ exonuclease activity^19^ and can be used for ORNi-PCR.^15^ We therefore examined whether *Pfu* DNA polymerase could also be used to detect genome-edited cells. As shown in Supplementary Fig. S5, ORN_20b or ORN_24b prevented *Pfu* DNA polymerase from amplifying WT gDNA, but not mutant gDNA. In contrast to ORNi-PCR using the KOD DNA polymerase, the ORN_Target had no suppressive effects (Supplementary Fig. S5A and B). Thus, *Pfu* DNA polymerase can also be used to detect genome-edited cells if an appropriate ORN is utilized.

One of the main aims of this study was to apply endpoint ORNi-PCR to detection of genome-edited cells. Nevertheless, it was also of interest to determine whether real-time (quantitative) ORNi-PCR is applicable for this purpose. To address this question, we performed ORNi-PCR on a real-time PCR machine. As shown in Supplementary Fig. S6, in real-time ORNi-PCR, PCR amplification of the *THYN1* locus from WT gDNA was inhibited in the presence of ORN_24b, whereas amplification from T4 and T6 gDNAs was not affected. In addition, with a template mimicking a mono-allelic indel mutation (WT + T4), amplification was suppressed by ∼60% in the presence of the ORN (Supplementary Fig. S6). In contrast, PCR amplification was not affected in the presence of an irrelevant ORN, ORN_302F(NC) (Supplementary Fig. S6). These results suggested that intact target sequences, mono-, and bi-allelic mutations can be discriminated by analysis of amplification patterns of real-time ORNi-PCR.

Genome editing can be performed using recombinant CRISPR ribonucleoproteins (RNPs)^20^^,^^21^. In this approach, synthesized sgRNAs or complexes of CRISPR RNAs (crRNAs) plus trans-activating crRNAs (tracrRNAs) are used as gRNAs. When performing genome editing using CRISPR RNPs, it would be more cost-effective to use crRNAs rather than ORNs in ORNi-PCR to detect genome-edited cells. Therefore, we tested the feasibility of using ORNi-PCR with crRNAs (Supplementary Fig. S7). The patterns of PCR amplification in the presence of crRNA_Target, a crRNA containing an RNA sequence complementary to the CRISPR target site (Supplementary Fig. S7A), were comparable to those obtained with *THYN1*-specific ORNs (Supplementary Fig. S7B and C and Figs 4 and 5). crRNA_NC, a crRNA targeting an irrelevant locus (chicken *Pax5* locus),^22^ did not affect amplification (Supplementary Fig. S7B and C). These results suggested that crRNAs can also be used for ORNi-PCR. In addition, an ORN containing irrelevant sequences in addition to a target-specific sequence could also be used for ORNi-PCR.

### 3.4 Screening of genome-edited cells by ORNi-PCR

**Figure 6.**
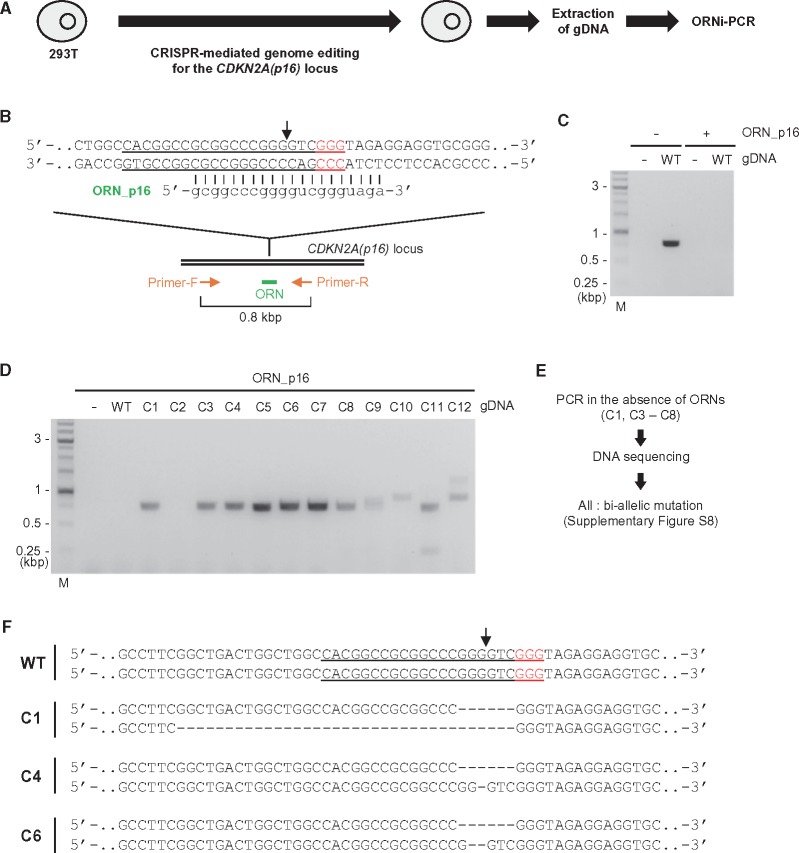
Screening of genome-edited cells by ORNi-PCR. (A) Schematic of screening of genome-edited cells by ORNi-PCR. (B) Target position of genome editing and sequence of a *CDKN2A(p16)*-specific ORN. The forward and reverse DNA sequences of the allele are shown. The CRISPR target site in 293T is underlined, and PAM positions are shown in red. A black arrow indicates the cleavage sites of CRISPR. (C) Setting of ORNi-PCR with gDNA extracted from 293T. M: molecular weight marker. (D) Results of ORNi-PCR (1 µM ORN). (E) Schematic of confirmation of indel mutations. (F) DNA sequences around the CRISPR target site in the *CDKN2A(p16)* locus in genome-edited cells. The forward DNA sequences of both alleles are shown.

Next, we applied ORNi-PCR to screening of genome-edited cells. Using CRISPR, we attempted to edit the *CDKN2A(p16)* locus in human 293T cells (Fig. 6A). After transfection of a CRISPR complex targeting this locus and single-colony isolation, gDNAs were extracted from 12 individual clones (C1–C12) and subjected to ORNi-PCR to detect genome-editing events. ORNi-PCR with ORN_p16, a *CDKN2A(p16)*-specific 20-base ORN (Fig. 6B and C), yielded amplification of the *CDKN2A(p16)* locus from 11 of the 12 samples (Fig. 6D), suggesting that genome editing had occurred in these clones. Two ORNi-PCR products were detected from the gDNAs of C9, C11, and C12, implying that these clones harbour distinct bi-allelic genome edits. The amplicon from C10 was of higher molecular weight (∼1 kb), suggesting that an insertional mutation occurred in this clone.

ORNi-PCR amplified 0.8 kb products from gDNAs of C1 and C3–C8 (Fig. 6D), the same size as the PCR product obtained when using WT gDNA as the template in the absence of ORNs (Fig. 6C). To characterize the types of mutations, we amplified the *CDKN2A(p16)* locus from gDNAs from clones with positive signals in ORNi-PCR in the absence of ORNs and directly sequenced the amplicons (Fig. 6E and Supplementary Fig. S8). No DNA-sequencing signals corresponding to the intact CRISPR target site were detected in the amplicons from C1 and C3–C8 (Supplementary Fig. S8C), suggesting that bi-allelic mutations had been introduced into the target sites in these clones. These results of DNA sequencing were consistent with those of ORNi-PCR. Thus, ORNi-PCR can be applied to screening of genome-edited cells. Notably in this regard, no DNA fragments were amplified from C2 gDNA even in the absence of ORNs (Supplementary Fig. S8B), potentially due to deletion of primer-binding sites, insertion of a DNA sequence too long to be amplified, or translocation of the *CDKN2A(p16)* locus.

At least three patterns of DNA-sequencing signals were detected in the amplicons of C3, C5, C7, and C8 (Supplementary Fig. S8C). Such mosaicism may have occurred in a step-by-step manner during genome editing (Supplementary Fig. S9), or have been caused by a mixture of different types of genome-edited cells (i.e. failed isolation of single-cell clones). Alternatively, it is also possible that such mosaicism was caused by aneuploidy in the cell cultures. In contrast, two patterns of DNA-sequencing signals were detected in the amplicons of C1, C4, and C6 (Fig. 6F and Supplementary Fig. S8C), suggesting that these cells were true clones of bi-allelically genome-edited cells.

### 3.5 Screening of genome-edited cells by multiplex ORNi-PCR

**Figure 7.**
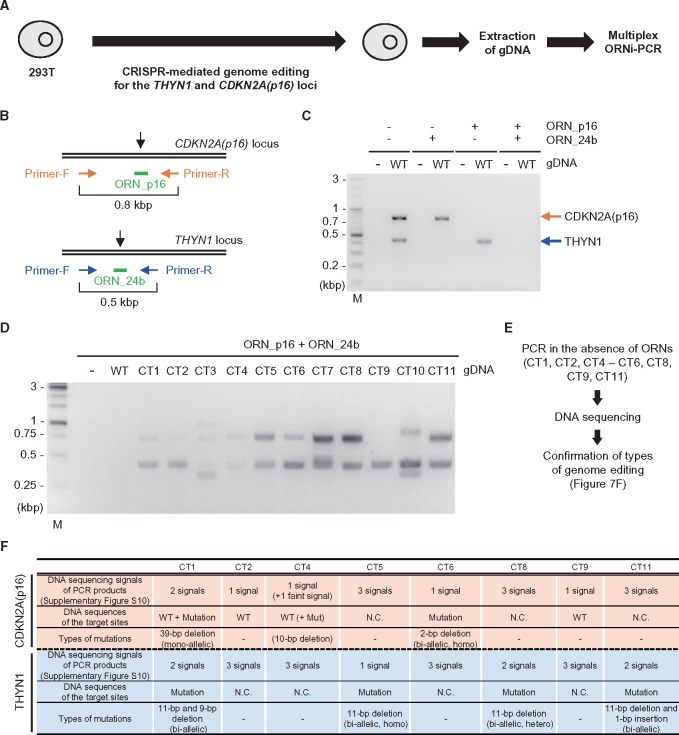
Screening of genome-edited cells by multiplex ORNi-PCR. (A) Schematic of screening of genome-edited cells by multiplex ORNi-PCR. (B) Target positions of ORNs. Black arrows indicate the cleavage sites of CRISPR. (C and D) Multiplex ORNi-PCR (1 µM each ORN) with gDNA extracted from 293T WT and genome-edited cells. Arrows indicate positions of PCR amplicons of *CDKN2A(p16)* (orange) and *THYN1* (blue). M, molecular weight marker. (E) Schematic of confirmation of indel mutations. (F) Types of indel mutations. N.C., not confirmed.

Genome editing can also occur simultaneously at multiple loci in a single cell. In this regard, it would be useful if genome-editing at multiple loci could be detected in a single-tube ORNi-PCR. We therefore examined whether multiplex ORNi-PCR can be used for screening of cells in which multiple target sites have been edited. Using CRISPR, we attempted to simultaneously edit the *CDKN2A(p16)* and *THYN1* loci in 293T cells (Fig. 7A). Multiplex ORNi-PCR with ORN_p16 and ORN_24b yielded PCR products from the *CDKN2A(p16)* locus from gDNAs of 9 out of 11 clones (CT1, CT3–CT8, CT10, and CT11), and from the *THYN1* locus from all gDNAs (Fig. 7B–D), suggesting that successful genome editing had occurred at each locus. The amplicons [*CDKN2A(p16)* and/or *THYN1*] of CT3, CT7, and CT10 were of different lengths, implying that the target loci were mutated mono-allelically or bi-allelically (Fig. 7D). To determine the types of indel mutations in the other samples, we amplified the *CDKN2A(p16)* and *THYN1* loci in the absence of ORNs and directly sequenced the amplicons (Fig. 7E and Supplementary Fig. S10). As summarized in Fig. 7F, CT2 and CT9 harboured mutations in the *THYN1* locus, but not in the *CDKN2A(p16)* locus, whereas the other clones harboured mutations in both loci. The types of genome editing (i.e. mono- or bi-allelic mutations) were confirmed in some samples (Fig. 7F and Supplementary Figs S10C and S11). The results of DNA sequencing were consistent with those of ORNi-PCR (Fig. 7D). Thus, multiplex ORNi-PCR can be applied to screening of cells in which multiple target sites have been edited.

### 3.6 Detection of point mutations by ORNi-PCR

**Figure 8.**
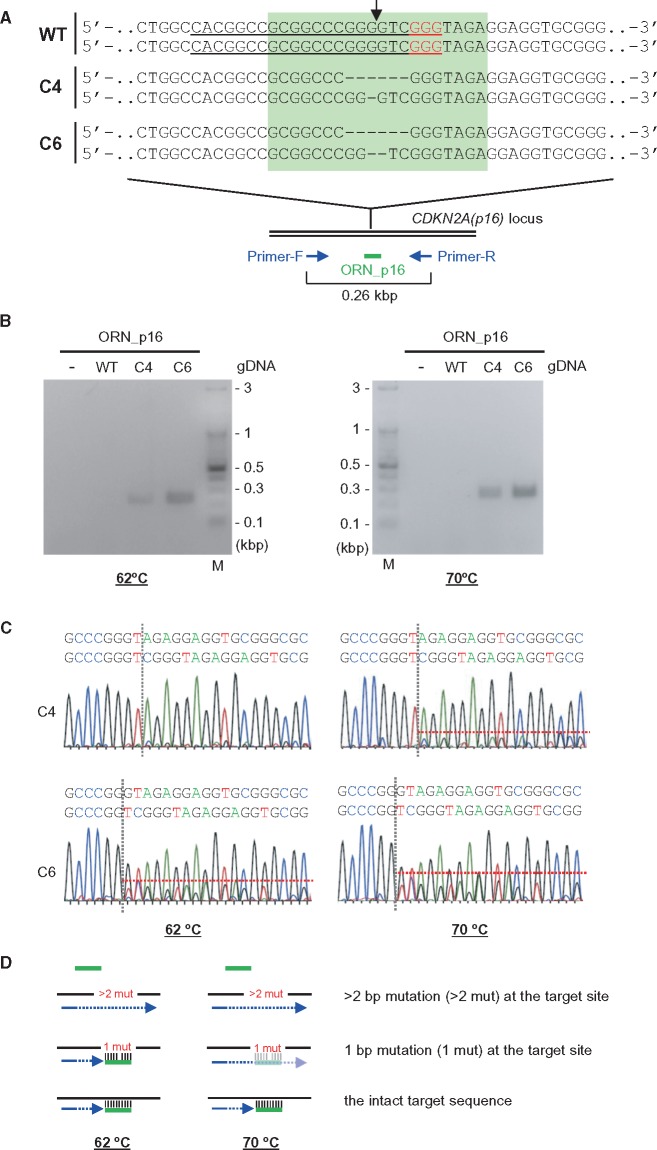
Discrimination of a single-nucleotide difference by ORNi-PCR. (A) Target positions of a *CDKN2A(p16)*-specific ORN, ORN_p16. Black arrow indicates the CRISPR cleavage sites. The forward DNA sequences around the CRISPR target site in the *CDKN2A(p16)* locus of both alleles are shown. The target site of the ORN is highlighted in light green. (B) Results of ORNi-PCR. PCR amplification was performed at two annealing temperatures (62 and 70°C) in the presence (1 µM) or absence of ORN_p16. M, molecular weight marker. (C) DNA-sequencing signals of ORNi-PCR products. ORNi-PCR products in (B) were purified from agarose gels and subjected to DNA sequencing using a forward primer. (D) Mode of discrimination of a single-nucleotide difference by ORNi-PCR. At an annealing temperature of 62°C, the ORN (green) hybridized with the target site containing a 1 bp mutation, but not a ≥2 bp mutation. Hybridization with the 1 bp-mutated target site was incomplete at an annealing temperature of 70°C, resulting in successful amplification. The ORN hybridized with the intact target site and strongly inhibited amplification at both temperatures.

Given that genome editing can be used to introduce a point mutation, and it is of practical importance to detect such mutations, we investigated whether ORNi-PCR is also capable of distinguishing point mutations. DNA sequencing of ORNi-PCR amplicons of C4 and C6 (Fig. 6D) yielded one and two patterns of DNA-sequencing signals, respectively (Supplementary Fig. S12). C4 and C6 possess 1 and 2 bp deletions, respectively, in the CRISPR target site in one allele (Fig. 6F). Thus, these results suggested that ORNi-PCR can detect a 2 bp deletion but not a 1 bp deletion of C4 under these experimental conditions (hybridization at 62°C, see Section 2). We next examined whether a higher hybridization temperature would enable detection of the 1 bp deletion of C4 (Fig. 8). To this end, we performed ORNi-PCR with ORN_p16 at an annealing temperature of 70 °C (Fig. 8A and B). Under these conditions, ORNi-PCR amplified the target product from C4, but not WT gDNA. Sequencing of the ORNi-PCR amplicon of C4 yielded a signal from the 1 bp deleted template. Similar conditions enabled detection of a 1 bp insertion (the *THYN1* locus in CT11, Supplementary Figs S11 and S13). Together, these results indicate that ORNi-PCR can distinguish a single-nucleotide indel mutation if the annealing temperature is optimized (Fig. 8D).

**Figure 9.**
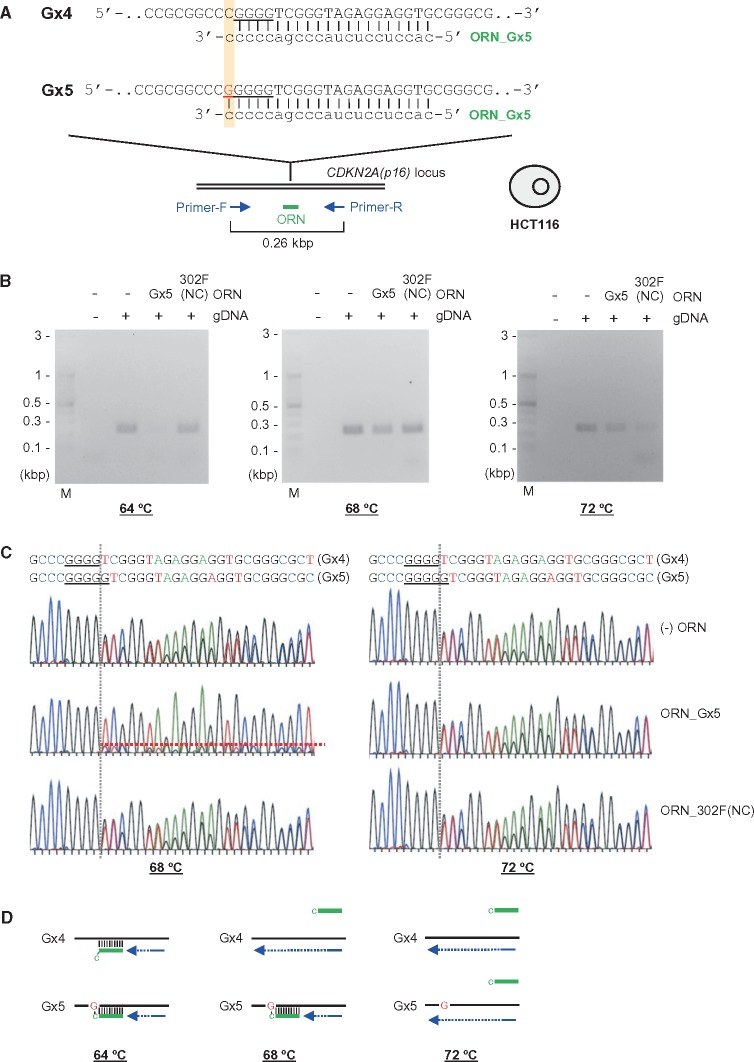
Detection of a point mutation by ORNi-PCR. Target position and sequence of a *CDKN2A(p16)*-specific ORN, ORN_Gx5. The forward DNA sequences of both alleles (Gx4 and Gx5) in HCT116 are shown. (B) Results of ORNi-PCR with gDNA extracted from HCT116. PCR was performed at various hybridization temperatures in the presence (1 µM) or absence of the ORN. M, molecular weight marker. (C) DNA-sequencing signals of PCR products. PCR products in (B) (68 and 72°C) were purified from agarose gels and subjected to DNA sequencing using a forward primer. (D) Mode of detection of point mutations by ORNi-PCR. (Left) At an annealing temperature of 64°C, ORN_Gx5 (green) hybridized with the target site in the Gx5 allele and the corresponding site in the Gx4 allele in the *CDKN2A(p16)* locus. (Middle) At an annealing temperature of 68°C, ORN_Gx5 hybridized with the target site in a Gx5 allele-specific manner. Because of a single-base mismatch of the 3′ cytosine of ORN_Gx5, the ORN cannot hybridize with the corresponding site in the Gx4 allele at this temperature. (Right) At an annealing temperature of 72°C, ORN_Gx5 did not hybridize even with the target sites.

We also investigated whether ORNi-PCR can detect point mutations in another sample. Here, we utilized the *CDKN2A*(*p16*) locus in human HCT116 cells as a model locus, in which an insertion of a single nucleotide (guanine) is present in the first exon in one allele (Fig. 9A).^17^^,^^23^ We designed an ORN, ORN_Gx5, complementary to the sequence of the G-insertion allele (Gx5) but with a single-nucleotide mismatch at the 3′ position with the corresponding DNA sequence in the other allele (Gx4) (Fig. 9A). As shown in Fig. 9B, ORN_Gx5 suppressed amplification of the *CDKN2A*(*p16*) locus when the annealing temperature was 64°C, suggesting that the ORN hybridized with the target DNA sequence in both alleles. In contrast, amplification was not clearly suppressed when the annealing temperature was raised to 68°C (Fig. 9B). Sequencing of the PCR product revealed that ORN_Gx5 suppressed PCR amplification of the *CDKN2A*(*p16*) locus from the Gx5 allele, but not the Gx4 allele (Fig. 9C). Such suppressive effects were not observed when the annealing temperature was raised to 72°C (Fig. 9B and C). Thus, these results provide further confirmation that ORNi-PCR can distinguish a point mutation if the annealing temperature is optimized (Fig. 9D).

Taken together, our findings show that ORNi-PCR can distinguish point mutations if the DNA/RNA hybridization is performed at the optimal temperature. Therefore, the optimal ORN hybridization temperature should be determined in advance for the detection of point mutations during the screening of genome-edited cells.

## 4. Discussion

In this study, we demonstrated that ORNi-PCR can be used to detect genome-edited cells without using expensive equipment such as real-time PCR machines. ORNi-PCR could discriminate an intact target DNA sequence from bi- and mono-allelic mutations (Figs 4 and 5). In addition, ORNi-PCR could be applied to screening of genome-edited cells (Figs 6 and 7). However, because it may be difficult to distinguish between mono- and bi-allelic mutations using endpoint ORNi-PCR, endpoint ORNi-PCR should be primarily used for the detection of genome-edited cells possessing at least mono-allelic mutations at a target site before subsequent determination of mono- or bi-allelic mutations by other methods (see detailed discussions on this below). In this study, we evaluated the introduction of CRISPR-mediated genome editing by ORNi-PCR. However, genome edits introduced by ZFNs or TALENs could also be detected by ORNi-PCR because the cleavage sites of these enzymes are also well defined.^24^

Mismatch cleavage assays using T7E1 or Surveyor nuclease are often used for the evaluation of indel mutations. In these approaches, after performing PCR of the target sequences, PCR products are denatured and reannealed, and DNA bulges at mismatch sites in the heteroduplexes are cleaved by these nucleases. These approaches are suitable for the evaluation of indel mutations in pools of genome-edited cells (i.e. in cells containing mixtures of WT and/or various types mutant sequences that easily form DNA bulges at mismatch sites). However, mismatch cleavage assays would not be suitable for screening of genome-edited cells because the addition of WT gDNA to the reaction would be required to detect a homozygous bi-allelic mutation in a single clone. In addition, it may be difficult to distinguish mono-allelic mutations from heterozygous bi-allelic mutations using this method. In this context, ORNi-PCR is more advantageous than mismatch cleavage assays (Table 1). On the other hand, because ORNi-PCR generates positive PCR signals when one allele of the genome of a target cell is edited, it may be difficult to distinguish bi- and mono-allelic mutations by endpoint ORNi-PCR. This feature is one of the drawbacks of ORNi-PCR not found in endpoint PCR-based methods^13^^,^^14^ (Table 1). Thus, endpoint ORNi-PCR should be primarily used to detect genome-edited cells possessing at least mono-allelic mutations at a target site before subsequent detection of mono- or bi-allelic mutations by other methods. However, endpoint ORNi-PCR would still be useful if hemi-knockout is also required, as in studies of X-chromosome inactivation, genomic imprinting, and cancer.^25–27^ In addition, ORNi-PCR could be applied when optimal primers cannot be designed in some PCR-based methods,^13^ especially as ORNs can be designed flexibly on their design.^15^ We demonstrated that genome-editing events at multiple loci can be detected in a single-tube endpoint ORNi-PCR (Fig. 7), which has not been achievable before using endpoint PCR-based methods.^13^^,^^14^ In this regard, the primer combination should be more carefully considered to avoid overlap of amplicons in the endpoint PCR-based methods.

**Table 1.** Comparison of genome-editing detection methods

Methods	Time (h)	Accuracy	Labor	Cost	Limitation
Mismatch cleavage assay^a^	>4	Moderate	Moderate	Moderate	Not suitable for detection of mutations that do not form DNA bulges at mismatches in the assay (e.g. homozygous bi-allelic mutations).
PCR-based method^a^	1–2	High	Low	Low	—
ORNi-PCR	1–2	High	Low	Low	It may be difficult to distinguish mono- and bi-allelic mutations by endpoint ORNi-PCR.

^a^Properties of these methods have been previously described.^13^

On the other hand, we also demonstrated that real-time ORNi-PCR could detect genome-edited cells (Supplementary Fig. S6) and distinguish between bi- and mono-allelic mutations. In real-time or digital PCR with fluorescent probes complementary to a target site of genome editing (e.g. TaqMan probes), the decline in fluorescent emission can be taken as an indication of genome-editing events.^10–12^ In contrast, the fluorescence emission of SYBR Green can be taken as an indication of genome-editing events in real-time ORNi-PCR. Notably, ORNi-PCR does not require modification of each ORN with fluorescent chemicals, representing an economic advantage over other detection methods that use fluorescent probes.

In our proof-of-principle experiments, genome-edited cells were efficiently isolated because of stable expression of the CRISPR complex (Figs 6 and 7, and see Section 2). Although this feature is useful for the characterization of genome editing performed by CRISPR, it is an undesirable feature in biological and medical applications. In such cases, transient expression of the CRISPR complex might be an optional strategy. However, sometimes large numbers of candidate clones must be screened if the efficiency of genome-editing is low, e.g. when the target region is not readily accessible to the genome-editing machinery, as in the case of heterochromatin. Because ORNi-PCR can detect various types of indel mutations (Figs 6–8), it would be useful for the detection of small numbers of genome-edited cells among large numbers of candidate cells.

We found that ORNi-PCR can distinguish point mutations if DNA/RNA hybridization is performed under optimal temperature (Figs 8 and 9 and Supplementary Fig. S13). This property of ORNi-PCR can also be used for detection of single-nucleotide polymorphisms (SNPs), mutations in pathogenic cells such as cancer cells, and other subtle differences in genome sequences. In this study, we estimated the hybridization temperatures of ORNs using a *T*_m_ calculation program, Oligo Calc^28^ (Supplementary Table S2), and annealing was performed at 62°C as a default setting in most ORNi-PCR experiments. This temperature was suitable for detection of ≥2 bp mutations (Figs 4, 8, and 9 and Supplementary Fig. S13). Several bases are usually edited in most cases of CRISPR-mediated genome editing in mammalian cells. In fact, we found ≥2 bp mutations in many cases (Figs 6 and 7). Thus, in the standard knock-out strategy, optimization of the annealing temperature might be unnecessary when screening genome-edited mammalian cells by ORNi-PCR. On the other hand, higher annealing temperatures were required to detect 1 bp mutations (Figs 8 and 9 and Supplementary Fig. S13). In this regard, if 1 bp mutations occurred in both alleles during genome editing, the method may have failed to detect such cells at an annealing temperature of 62°C. To avoid such false negatives, it is recommended to empirically determine the optimal hybridization temperature in advance by performing ORNi-PCR on WT gDNA. To distinguish single-nucleotide differences, it may be preferable to use the highest temperature at which the ORN can hybridize with the target sequence.

PCR can be perturbed by unpredicted contaminants when impure gDNAs are used. In such cases, it would be difficult to judge whether the absence of amplification in ORNi-PCR reflects sequence-specific suppression by ORNs or such an undesirable perturbation. In this regard, it would be useful to amplify an irrelevant locus as an internal control in parallel with ORNi-PCR in the same reaction mixture; amplification of the internal control would indicate that the reaction conditions were suitable for PCR. In fact, we showed that it was possible to evaluate introduction of genome editing using such a multiplex PCR system, in which a target primer set, a target-specific ORN, and another primer set that amplified an internal control were mixed in the same tube (Supplementary Fig. S14). These results also ensure target specificity of the designed ORNs, and ORNi-PCR with an internal control PCR could more reliably detect genome-editing events.

In this study, we used ORNs at 1 μM to screen genome-edited cells (see Section 2) because PCR amplification of a target locus in WT gDNA was completely suppressed by ORNs at 1 or 2 μM (Fig. 2B). Lower concentrations of some ORNs were less effective and resulted in the appearance of faint amplicons (e.g. with ORN_20b at 0.1 or 0.5 μM in Fig. 2B). Because such amplicons might lead to false positive results, effective concentrations of ORNs should be determined by titration as shown in Fig. 2. Additionally, we used 20 ng purified human gDNAs in 10 µl reaction mixtures for 30–35 cycles of ORNi-PCR. ORN suppression might be insufficient if excess amounts of gDNAs or more cycles are used, and might also skew the results. Concerning the DNA purification method, we used gDNAs purified by the standard phenol/chloroform extraction, which could be laborious when many clones need to be handled. Crude extracts might provide an easier option, but potential RNase activity in the crude extracts could degrade ORNs in the ORNi-PCR reactions. However, this could be avoided by adding RNase inhibitors to the reactions. To increase the flexibility of ORNi-PCR, the ORNi-PCR protocol should be further optimized in the future.

In summary, we showed that ORNi-PCR can be applied to screening of genome-edited cells without expensive equipment. We believe that, despite its drawbacks, ORNi-PCR is a potentially useful tool that will make genome editing easier. In addition, ORNi-PCR enables detection of SNPs, mutations in pathogenic cells such as cancer cells, and other subtle differences in genome sequences.

## Supplementary Material

Supplementary Figures S1-S14

Supplementary Table S1

Supplementary Table S2

Supplementary Text
