# Supplementary material for: Detection of genome-edited cells by oligoribonucleotide interference-PCR
Source: DNA Res. 2018 Apr 27;25(4):395–407. doi: 10.1093/dnares/dsy012 (PMC6105111; doi:10.1093/dnares/dsy012)
Supplement: Supplementary Figures S1-S14 [file dsy012_supplementary_figures_s1-s14.pdf]

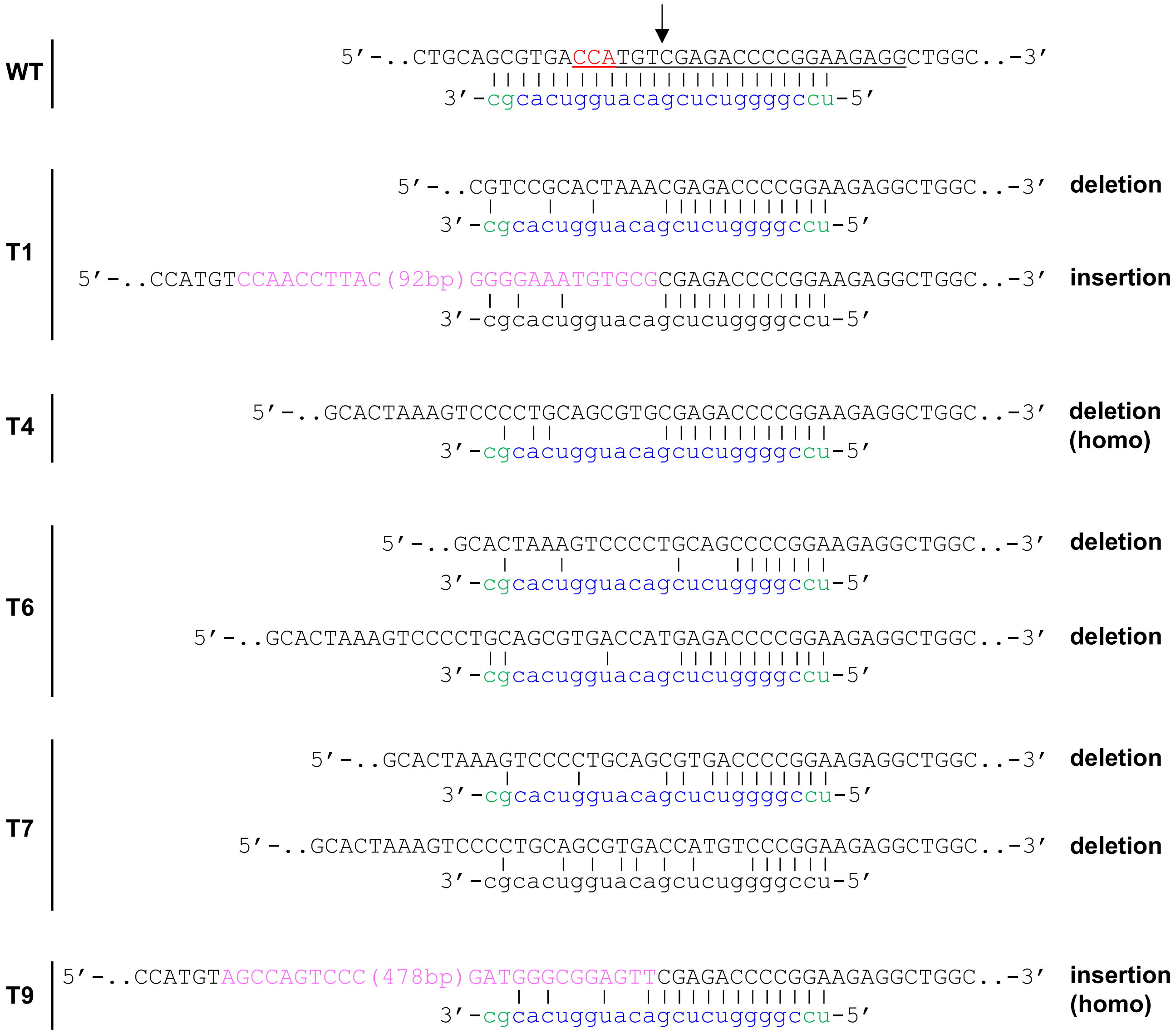

Supplementary Figure S1

```
WT     5′-..CTGCAGCGTGACCATGTCGAGACCCCGGAAGAGGCTGGC..-3′
                    3′-gguacagcucuggggccuucucc-5′

T1     5′-..CGTCCGCACTAAACGAGACCCCGGAAGAGGCTGGC..-3′        deletion
                 3′-gguacagcucuggggccuucucc-5′

       5′-..CCATGTCCAACCTTAC(92bp)GGGGAAATGTGCGCGAGACCCCGGAAGAGGCTGGC..-3′    insertion
                          3′-gguacagcucuggggccuucucc-5′

T4     5′-..GCACTAAAGTCCCCTGCAGCGTGCGAGACCCCGGAAGAGGCTGGC..-3′    deletion
                      3′-gguacagcucuggggccuucucc-5′             (homo)

T6     5′-..GCACTAAAGTCCCCTGCAGCCCCGGAAGAGGCTGGC..-3′      deletion
              3′-gguacagcucuggggccuucucc-5′

       5′-..GCACTAAAGTCCCCTGCAGCGTGACCATGAGACCCCGGAAGAGGCTGGC..-3′    deletion
                           3′-gguacagcucuggggccuucucc-5′

T7     5′-..GCACTAAAGTCCCCTGCAGCGTGACCCCGGAAGAGGCTGGC..-3′    deletion
                  3′-gguacagcucuggggccuucucc-5′

       5′-..GCACTAAAGTCCCCTGCAGCGTGACCATGTCCCGGAAGAGGCTGGC..-3′    deletion
                      3′-gguacagcucuggggccuucucc-5′

T9     5′-.. CCATGTAGCCAGTCCC(478bp)GATGGGCGGAGTTCGAGACCCCGGAAGAGGCTGGC..-3′    insertion
                          3′-gguacagcucuggggccuucucc-5′                            (homo)
```

Supplementary Figure S2

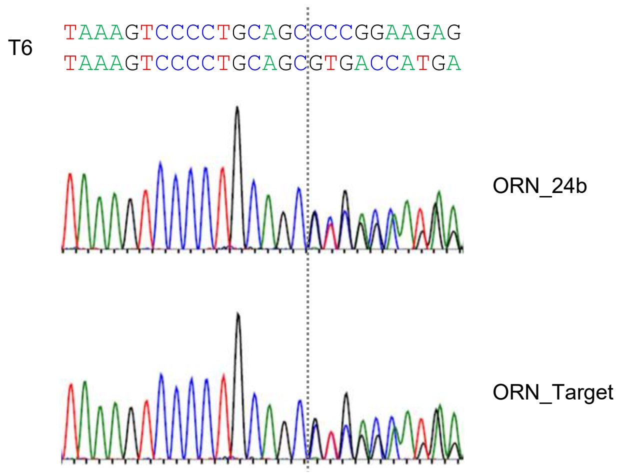

Supplementary Figure S3

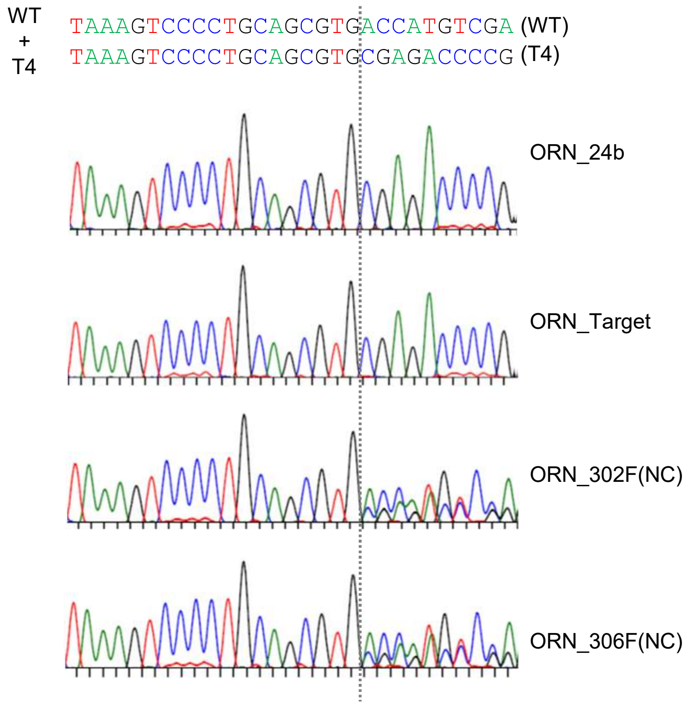

Supplementary Figure S4

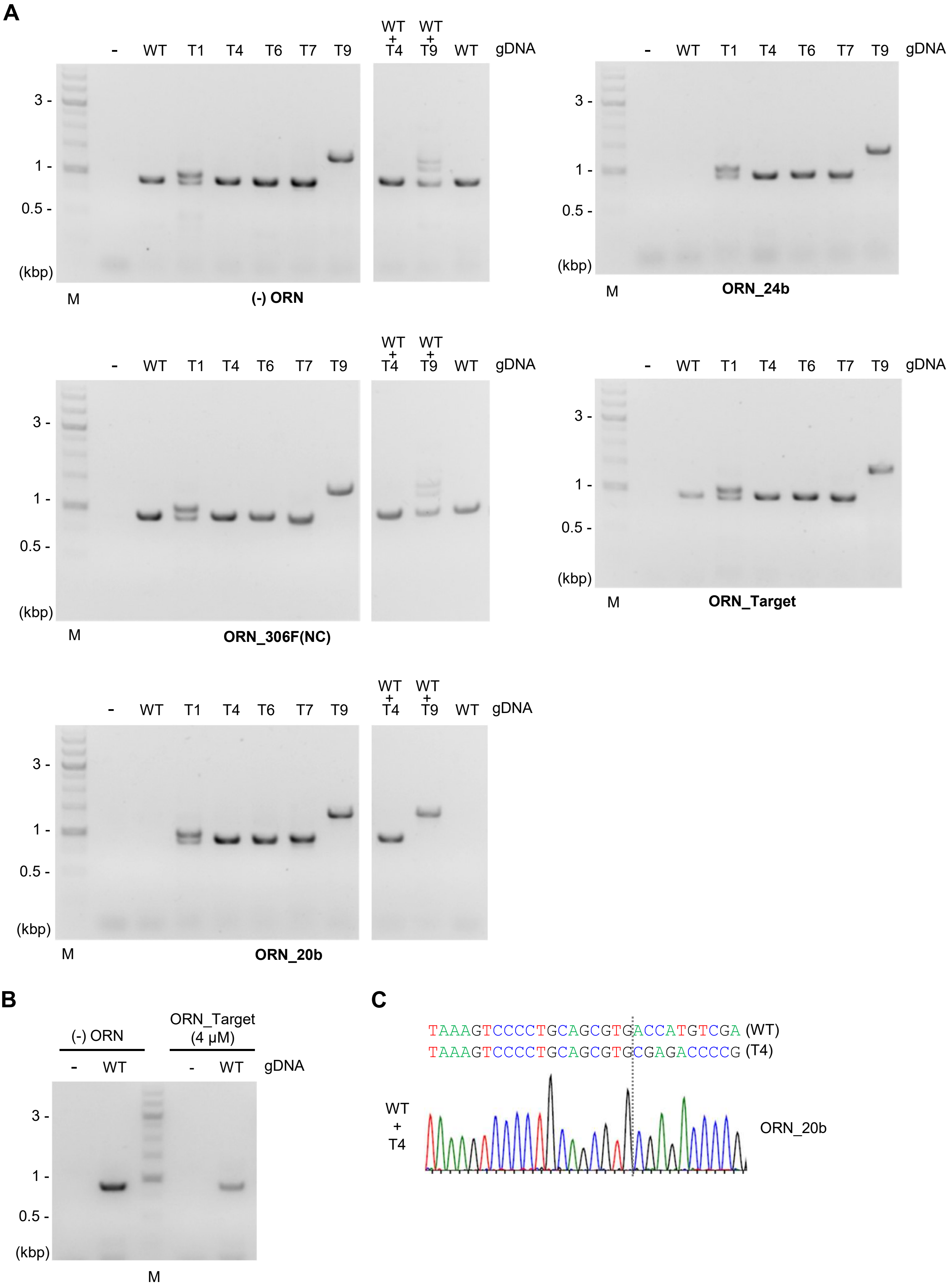

Supplementary Figure S5

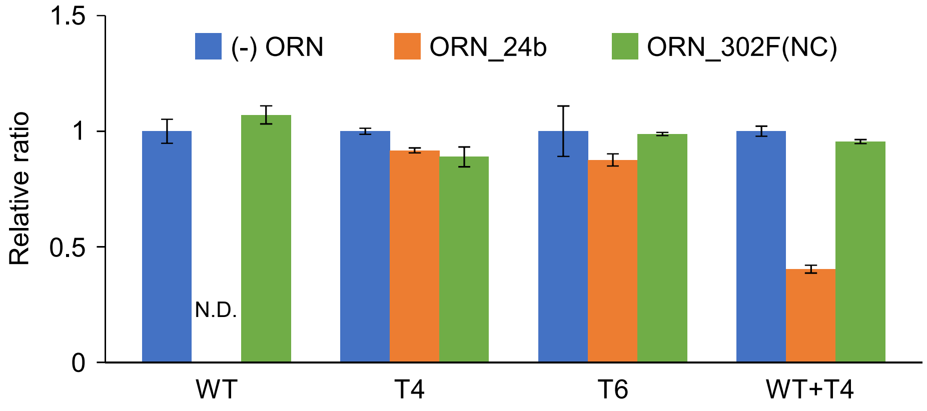

Supplementary Figure S6

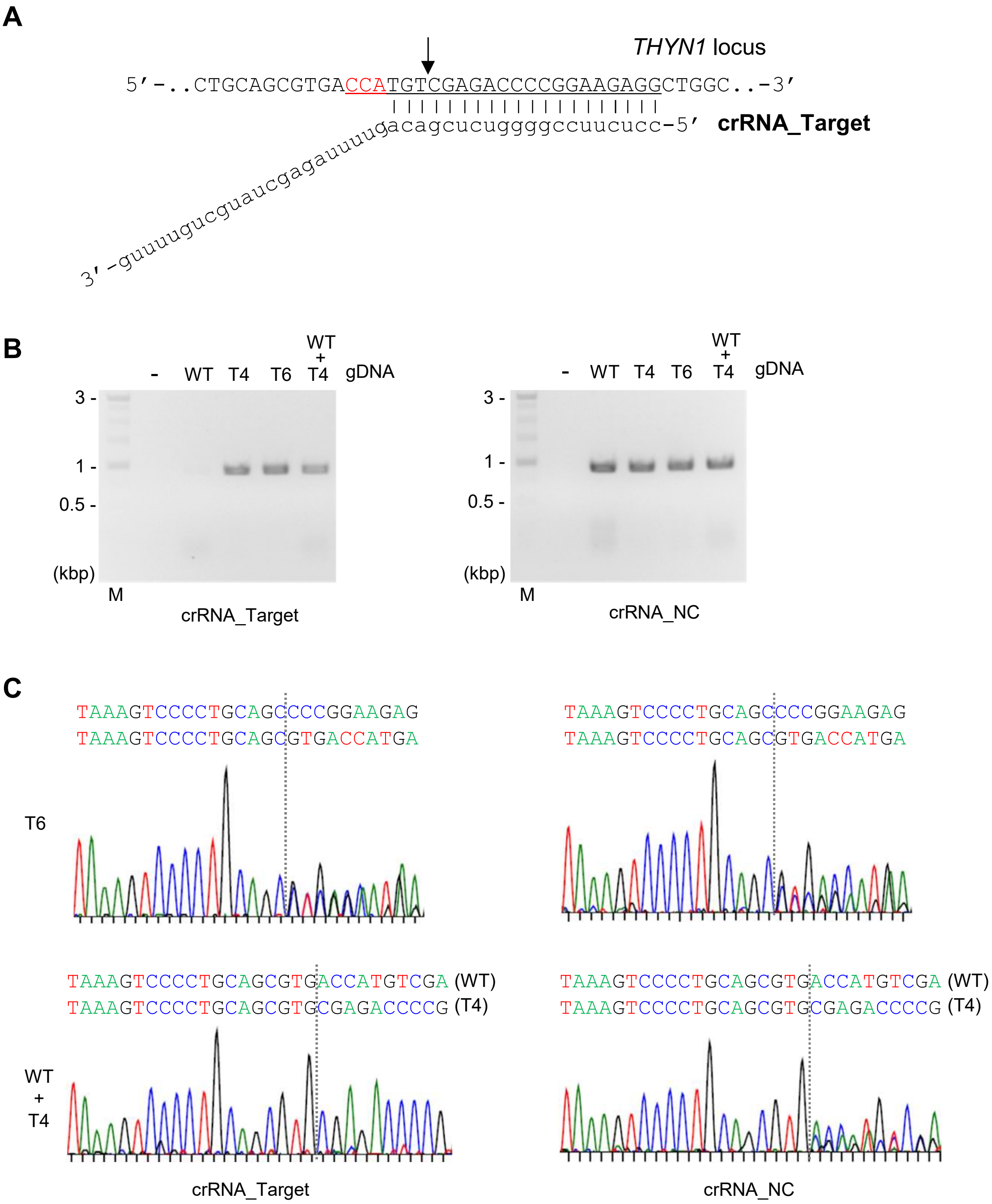

Supplementary Figure S7

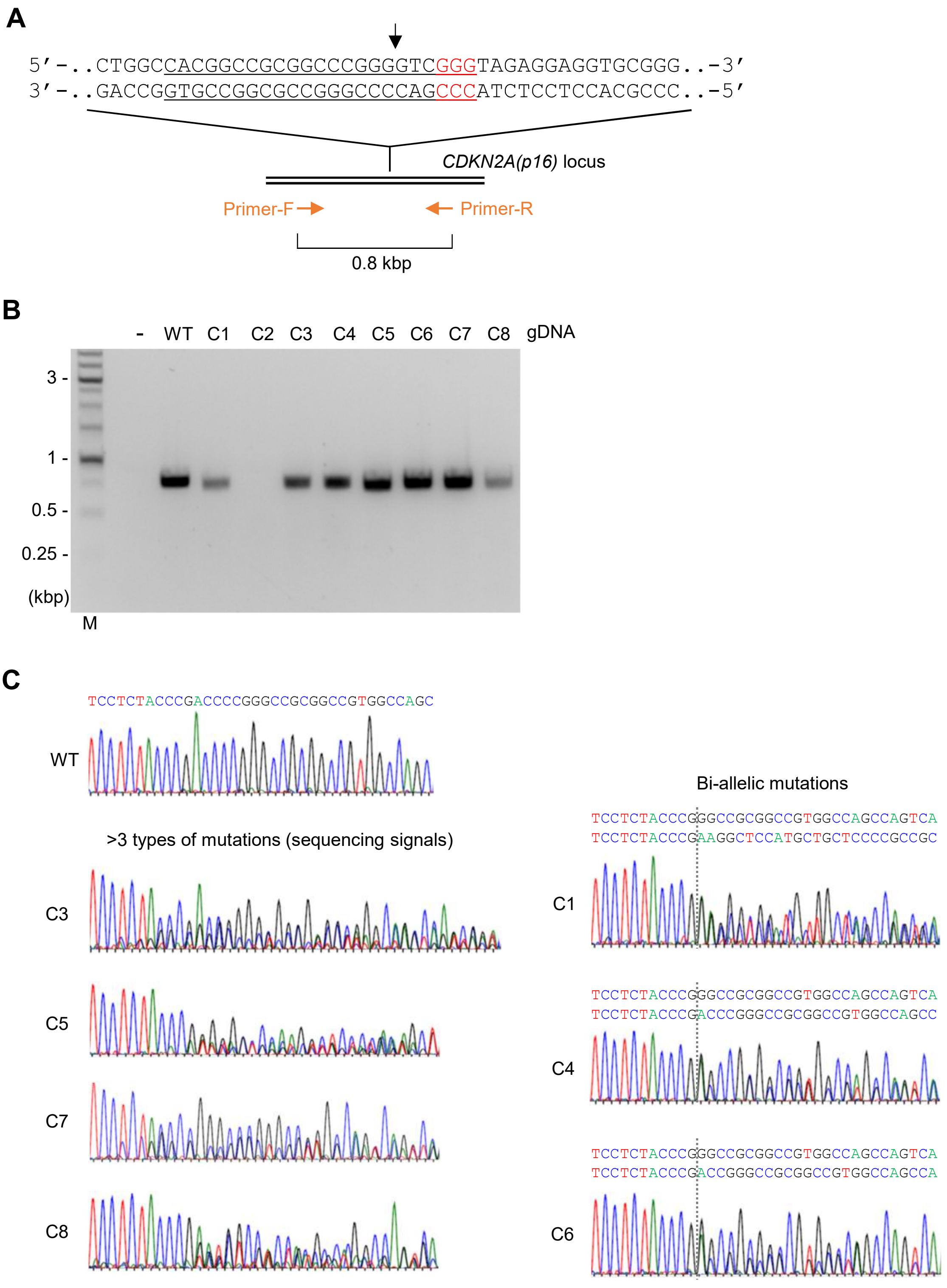

Supplementary Figure S8

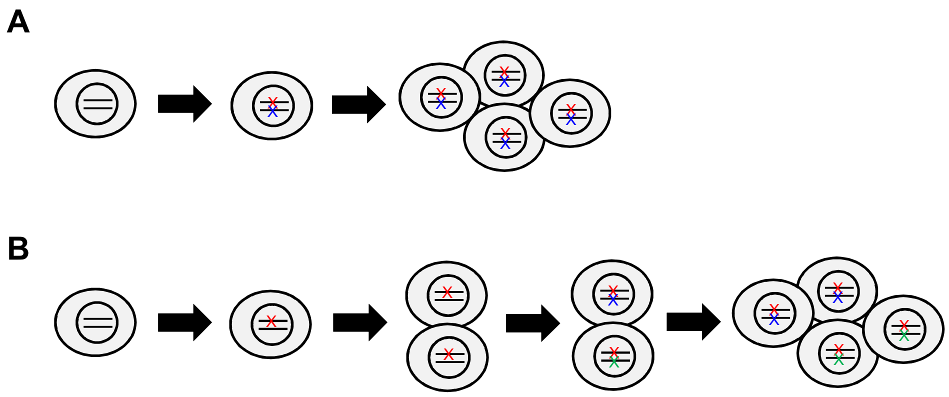

Supplementary Figure S9

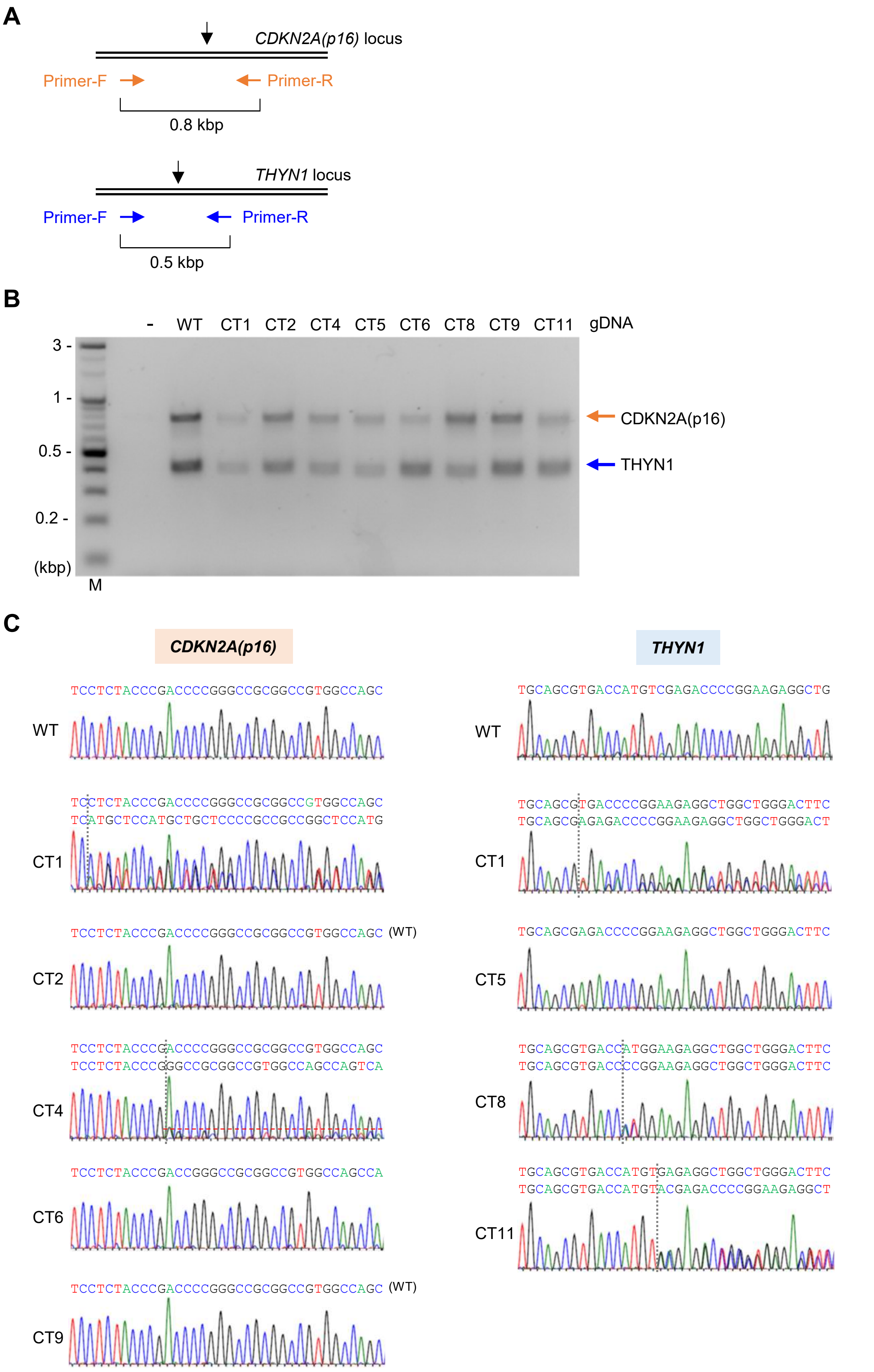

Supplementary Figure S10

CDKN2A(p16)

```
WT    5′-..GCCTTCGGCTGACTGGCTGGCCACGGCCGCGGCCCGGGGTCGGGTAGAGGAGGTGC..-3′
      5′-..GCCTTCGGCTGACTGGCTGGCCACGGCCGCGGCCCGGGGTCGGGTAGAGGAGGTGC..-3′

CT1   5′-..GCCTTCGGCTGACTGGCTGGCCACGGCCGCGGCCCGGGGTCGGGTAGAGGAGGTGC..-3′  deletion
      5′-..GCAT---------------------------------------------GAGGTGC..-3′  deletion

CT4   5′-..GCCTTCGGCTGACTGGCTGGCCACGGCCGCGGCCCGGGGTCGGGTAGAGGAGGTGC..-3′
      5′-..GCCTTCGGCTGACTGGCTGGCCACGGCCGCGGCC------CGGGTAGAGGAGGTGC..-3′  deletion

CT6   5′-..GCCTTCGGCTGACTGGCTGGCCACGGCCGCGGCCCG--GTCGGGTAGAGGAGGTGC..-3′  deletion
      5′-..GCCTTCGGCTGACTGGCTGGCCACGGCCGCGGCCCG--GTCGGGTAGAGGAGGTGC..-3′  deletion
```

THYN1

```
WT    5′-..GCACTAAAGTCCCCTGCAGCGTGACCATGTCGAGACCCCGGAAGAGGCTGGC..-3′
      5′-..GCACTAAAGTCCCCTGCAGCGTGACCATGTCGAGACCCCGGAAGAGGCTGGC..-3′

CT1   5′-..GCACTAAAGTCCCCTGCAGCGT-----------GACCCCGGAAGAGGCTGGC..-3′ deletion
      5′-..GCACTAAAGTCCCCTGCAGCG---------AGAGACCCCGGAAGAGGCTGGC..-3′ deletion

CT5   5′-..GCACTAAAGTCCCCTGCAGCG-----------AGACCCCGGAAGAGGCTGGC..-3′ deletion
      5′-..GCACTAAAGTCCCCTGCAGCG-----------AGACCCCGGAAGAGGCTGGC..-3′ deletion

CT8   5′-..GCACTAAAGTCCCCTGCAGCGTGACCAT-----------GGAAGAGGCTGGC..-3′ deletion
      5′-..GCACTAAAGTCCCCTGCAGCGTGA-----------CCCCGGAAGAGGCTGGC..-3′ deletion

CT11  5′-..GCACTAAAGTCCCCTGCAGCGTGACCATGT----------G-AGAGGCTGGC..-3′  deletion
      5′-..GCACTAAAGTCCCCTGCAGCGTGACCATGTCGAGACCCCGGAAGAGGCTGGC..-3′  insertion
                                       ^
                                       A
```

Supplementary Figure S11

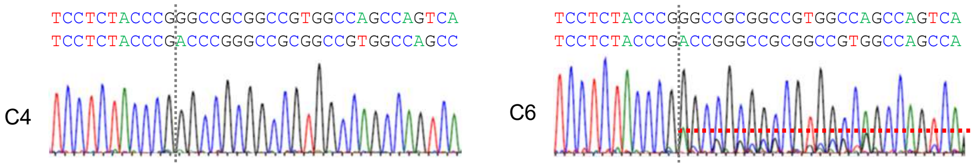

Supplementary Figure S12

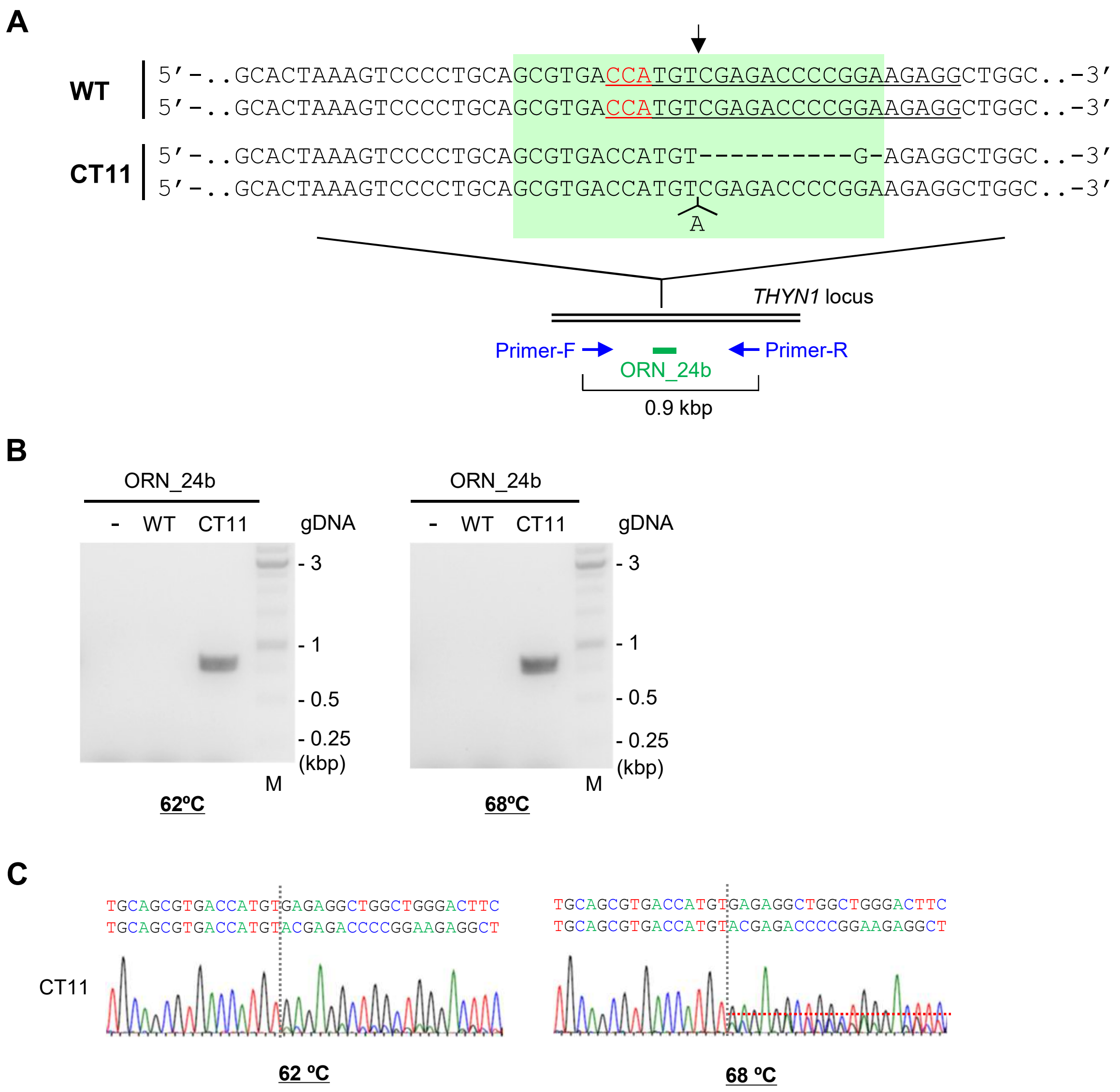

Supplementary Figure S13

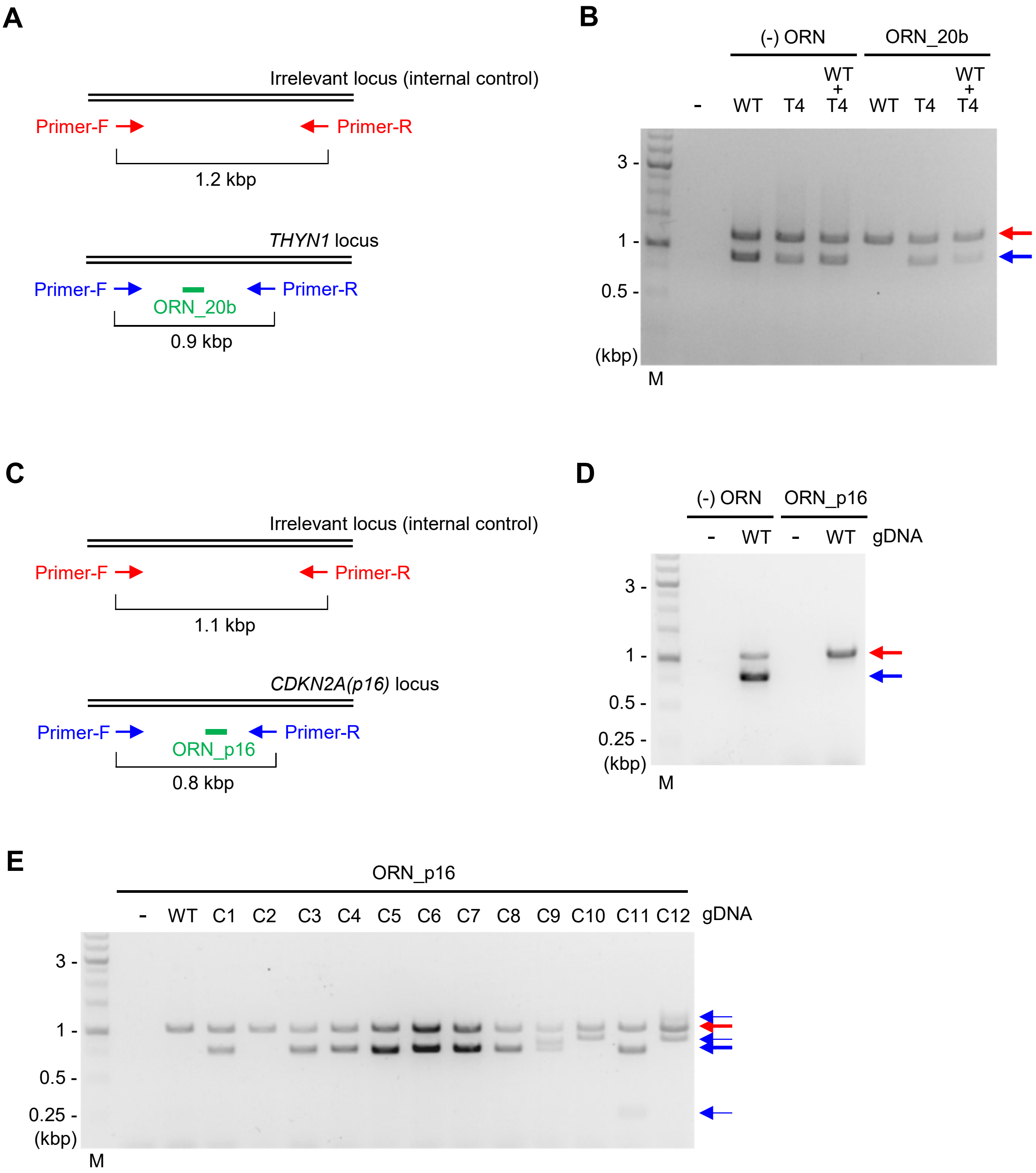

Supplementary Figure S14
