# Supplementary material for: Detection of genome-edited cells by oligoribonucleotide interference-PCR
Source: DNA Res. 2018 Apr 27;25(4):395–407. doi: 10.1093/dnares/dsy012 (PMC6105111; doi:10.1093/dnares/dsy012)
Supplement: Supplementary Text [file dsy012_supplementary_text.doc]

**Supplementary Text**

**Detection of genome-edited cells by oligoribonucleotide (ORN) interference-PCR (ORNi-PCR)**

Toshitsugu Fujita, Miyuki Yuno, Fusako Kitaura, and Hodaka Fujii

**Supplementary Table and Figure Legends**

**Supplementary Table S1. Primers used in this study**

**Supplementary Table S2. ORNs and crRNAs used in this study**

**Supplementary Figure S1. DNA sequences targeted by ORN_20b or ORN_24b**

The forward DNA sequences around the ORN target sites in WT and genome-edited Raji cells are shown. ORN_20b and ORN_24b are shown in blue and blue plus green, respectively (See also Figure 2A). The CRISPR target site in WT is underlined, and the PAM position is shown in red. An arrow indicates the CRISPR cleavage sites. Inserted DNA sequences in T1 and T9 are shown in pink. Only one allele is shown for WT, T4, and T9.

**Supplementary Figure S2. DNA sequences targeted by ORN_Target**

The forward DNA sequences around the ORN target sites in WT and genome-edited Raji cells are shown. ORN_Target is shown in green (See also Figure 2A). The CRISPR target site in WT is underlined, and the PAM position is shown in red. An arrow indicates the CRISPR cleavage sites. Inserted DNA sequences in T1 and T9 are shown in pink. Only one allele is shown for WT, T4, and T9.

**Supplementary Figure S3. DNA sequencing signals of PCR products**

PCR products of T6 (ORN_24b and ORN_Target) in Figure 4C were purified from agarose gels and subjected to DNA sequencing using a forward primer.

**Supplementary Figure S4. DNA sequencing signals of ORNi-PCR products**

PCR was performed with WT plus T4 gDNAs, which mimic a mono-allelic mutation, in the presence (1 µM) of ORNs. The ORNi-PCR products were purified and subjected to DNA sequencing using a forward primer. The DNA sequencing signals are shown here.

**Supplementary Figure S5. ORNi-PCR using *Pfu* DNA polymerase**

(**A**) ORNi-PCR with gDNAs extracted from WT and genome-edited Raji cells. PCR was performed with *Pfu* DNA polymerase in the presence (1 μM) or absence of ORNs. M: molecular weight marker. (**B**) ORNi-PCR with ORN_Target (4 μM). (**C**) DNA sequencing signals of a PCR product. The PCR product of WT + T4 (ORN_20b) in (**A**) was purified from agarose gels and subjected to DNA sequencing using a forward primer.

**Supplementary Figure S6. Results of real-time (quantitative) ORNi-PCR**

Real-time PCR was performed in the presence (0.25 μM) or absence of ORNs. The error bars represent the range of technical duplicates. Magnitudes of PCR amplification of the controls [(-) ORN] were defined as 1. N.D.: Not Detected.

**Supplementary Figure S7. ORNi-PCR using a crRNA**

(**A**) Target position and sequence of crRNA_Target. The forward DNA sequence of the allele is shown. The CRISPR target site in WT Raji is underlined, and the PAM position is shown in red. An arrow indicates the CRISPR cleavage sites. (**B**) crRNA-mediated inhibition of PCR amplification. PCR was performed in the presence (2 μM) or absence of crRNAs. crRNA_NC, a crRNA targeting an irrelevant locus (chicken *Pax5*), was used as a negative control. M: molecular weight marker. (**C**) DNA sequencing signals of PCR products. The PCR products of T6 and WT + T4 in (**B**) were purified from agarose gels and subjected to DNA sequencing using a forward primer.

**Supplementary Figure S8. PCR to detect indel mutations in the *CDKN2A(p16)* locus**

(**A**) Experimental scheme of PCR. The CRISPR target site in 293T is underlined, and the PAM is shown in red. A black arrow indicates the CRISPR cleavage sites. (**B**) Results of PCR. PCR was performed with gDNAs extracted from WT and genome-edited 293T. (**C**) DNA sequencing signals of the PCR products. The PCR products of WT, C1, and C3–C8 in (**B**) were purified from agarose gels and subjected to DNA sequencing using a reverse primer.

**Supplementary Figure S9. Scheme of potential modes of genome editing**

(**A**) Genome editing is introduced in a bi-allelic manner in a single cell, which is expanded as a genome-edited clone. (**B**) The first genome edit is introduced in a mono-allelic manner. The second genome edit is introduced into the other allele after division, which results in mosaicism of cells.

**Supplementary Figure S10. Multiplex PCR to confirm indel mutations**

(**A**) Experimental scheme of multiplex PCR. Black arrows indicate the CRISPR cleavage sites. (**B**) Results of multiplex PCR. Multiplex PCR was performed with gDNAs extracted from WT and genome-edited 293T. (**C**) DNA sequencing signals of the PCR products. The PCR products in (**B**) were purified from agarose gels and subjected to DNA sequencing using a reverse primer [*CDKN2A(p16)*] and a forward primer (*THYN1*). See also Supplementary Figure S10. Results not shown here [CT5, CT8, and CT11 of *CDKN2A(p16)* and CT2, CT4, CT6, and CT9 of *THYN1*] revealed three DNA sequencing signals, implying mosaicism.

**Supplementary Figure S11. DNA sequences around the CRISPR target sites in WT and genome-edited 293T**

The forward DNA sequences of both alleles are shown. The CRISPR target sites in 293T are underlined, and PAM positions are shown in red. Arrows show the cleavage sites of CRISPR. See also Figure 8 and Supplementary Figure S9.

**Supplementary Figure S12. DNA sequencing signals of ORNi-PCR products**

The ORNi-PCR products of C4 and C6 in Figure 6D were purified from gels and subjected to DNA sequencing using a reverse primer.

**Supplementary Figure S13. Discrimination of a single-nucleotide difference by ORNi-PCR**

(**A**) Target positions of a *THYN1*-specific ORN, ORN_24b. Black arrow indicates the CRISPR cleavage sites. The forward DNA sequences around a CRISPR target site in the *THYN1* locus of both alleles are shown. The target site of the ORN is highlighted in light green. (**B**) Results of ORNi-PCR. PCR amplification was performed at two annealing temperatures (62°C and 68°C) in the presence (1 µM) or absence of ORN_24b. M: molecular weight marker. (**C**) DNA sequencing signals of ORNi-PCR products. ORNi-PCR products in (**B**) were purified from agarose gels and subjected to DNA sequencing using a forward primer.

**Supplementary Figure S14. ORNi-PCR in combination with an internal control PCR**

(**A**) Experimental scheme of ORNi-PCR for the *THYN1* locus in combination with PCR for an irrelevant locus (i.e., an internal control). The *PD-L1* locus was amplified as an internal control. (**B**) Results of ORNi-PCR in combination with internal control PCR. PCR was performed with gDNAs extracted from WT and genome-edited Raji cells in the presence (1 μM) or absence of ORN_20b. M: molecular weight marker. (**C**) Experimental scheme of ORNi-PCR for the *CDKN2A(p16)* locus in combination with PCR for an irrelevant locus (internal control). The *Cyclin D1* locus was amplified by PCR as an internal control. (**D**) Setting of ORNi-PCR in combination with an internal control PCR. PCR was performed with gDNA extracted from 293T in the presence (1 μM) or absence of ORN_p16. (**E**) Results of ORNi-PCR in combination with an internal control PCR. PCR was performed with gDNAs extracted from WT and genome-edited 293T in the presence (1 μM) or absence of ORN_p16.

**Supplementary Materials and Methods**

**ORNi-PCR using *Pfu* DNA polymerase**

ORNi-PCR reactions were performed using Pfu-X (Jena). Briefly, ORNi-PCR reaction mixtures containing 20 ng of gDNA, 0.4 μM of each primer, and 1 or 4 μM of an ORN were prepared in a 10 μl volume according to the manufacturer’s protocol. The reactions were carried out with an initial denaturation at 95°C for 2 min; 35 cycles of 95°C for 20 sec, 62°C for 30 sec, and 68°C for 1 min; and final extension at 68°C for 1 min. ORNi-PCR products were electrophoresed on 1% agarose gels.

**Real-time ORNi-PCR**

Real-time ORNi-PCR reactions were performed using KOD SYBR qPCR Mix (Toyobo). Real-time ORNi-PCR reaction mixtures containing 20 ng of gDNA, 0.2 μM of each primer, and 0.25 μM of an ORN were prepared in 10 μl reactions according to the manufacturer’s protocol. The reactions were carried out with an initial denaturation at 98°C for 2 min, followed by 30 cycles of denaturation at 98°C for 10 sec, primer annealing at 62°C for 30 sec, and extension at 68°C for 1 min. ORNi-PCR amplification was quantitated on a 7900HT Fast Real-Time PCR System (Applied Biosystems). A unique ORNi-PCR amplicon of the expected size was confirmed by electrophoresis on 1% agarose gels.

**ORNi-PCR with a crRNA**

ORNi-PCR reactions were performed using KOD-Plus-Ver.2 (Toyobo) as described in Materials and Methods, except that 2 μM crRNA was used.

**ORNi-PCR for detection of a point mutation in the *THYN1* locus**

ORNi-PCR reaction mixtures were prepared using KOD-Plus-Ver.2 (Toyobo) as described in Materials and Methods. The reactions were carried out with an initial denaturation at 94°C for 2 min, followed by 30 cycles of 98°C for 10 sec, 62 or 68°C for 30 sec, and 68°C for 1 min.

**ORNi-PCR with an internal control PCR**

ORNi-PCR reactions were performed using KOD-Plus-Ver.2 (Toyobo) as described in Materials and Methods, except for addition of a primer set for the internal control PCR.
